# Anxiolytic, Analgesic and Anti-Inflammatory Effects of Peptides Hmg 1b-2 and Hmg 1b-4 from the Sea Anemone *Heteractis magnifica*

**DOI:** 10.3390/toxins15050341

**Published:** 2023-05-15

**Authors:** Irina N. Gladkikh, Anna A. Klimovich, Rimma S. Kalina, Yulia V. Kozhevnikova, Timur A. Khasanov, Dmitry I. Osmakov, Sergey G. Koshelev, Margarita M. Monastyrnaya, Yaroslav A. Andreev, Elena V. Leychenko, Sergey A. Kozlov

**Affiliations:** 1 G.B. Elyakov Pacific Institute of Bioorganic Chemistry, Far Eastern Branch, Russian Academy of Sciences, 690022 Vladivostok, Russia; annaklim_1991@mail.ru (A.A.K.); kalinarimma@gmail.com (R.S.K.); yliya77ya@mail.ru (Y.V.K.); rita1950@mail.ru (M.M.M.); leychenko@gmail.com (E.V.L.); 2 Shemyakin-Ovchinnikov Institute of Bioorganic Chemistry, Russian Academy of Sciences, 117997 Moscow, Russia; hasanov.ta@phystech.edu (T.A.K.); osmadim@gmail.com (D.I.O.); sknew@yandex.ru (S.G.K.); aya@ibch.ru (Y.A.A.); serg@ibch.ru (S.A.K.); 3 Institute of Molecular Medicine, Sechenov First Moscow State Medical University, 119991 Moscow, Russia

**Keywords:** sea anemones (*Heteractis magnifica*), APETx-like peptides, acid-sensing ion channels, anxiolytic effect, pain killers, inflammation

## Abstract

Acid-sensing ion channels (ASICs) have been known as sensors of a local pH change within both physiological and pathological conditions. ASIC-targeting peptide toxins could be potent molecular tools for ASIC-manipulating in vitro, and for pathology treatment in animal test studies. Two sea anemone toxins, native Hmg 1b-2 and recombinant Hmg 1b-4, both related to APETx-like peptides, inhibited the transient current component of human ASIC3-Δ20 expressed in *Xenopus laevis* oocytes, but only Hmg 1b-2 inhibited the rat ASIC3 transient current. The Hmg 1b-4 action on rASIC3 as a potentiator was confirmed once again. Both peptides are non-toxic molecules for rodents. In open field and elevated plus maze tests, Hmg 1b-2 had more of an excitatory effect and Hmg 1b-4 had more of an anxiolytic effect on mouse behavior. The analgesic activity of peptides was similar and comparable to diclofenac activity in an acid-induced muscle pain model. In models of acute local inflammation induced by λ-carrageenan or complete Freund’s adjuvant, Hmg 1b-4 had more pronounced and statistically significant anti-inflammatory effects than Hmg 1b-2. It exceeded the effect of diclofenac and, at a dose of 0.1 mg/kg, reduced the volume of the paw almost to the initial volume. Our data highlight the importance of a comprehensive study of novel ASIC-targeting ligands, and in particular, peptide toxins, and present the slightly different biological activity of the two similar toxins.

## 1. Introduction

Inflammation is associated with numerous physical illnesses in mammalians, including arthritis, asthma, atherosclerosis, diabetes, cancer, and autoimmune and neurodegenerative diseases [1]. The releasing of inflammatory mediators during inflammation, such as cytokines and chemokines, prostaglandins, leukotrienes, histamine, adenosine triphosphate, reactive oxygen species and protons, could activate both immune and neuronal cells [2]. Under the control of these inflammatory mediators, the expression and functioning of nociceptive ion channels in sensory neurons could be altered, which results in the overall excitability of peripheral nociceptive fibers, thus causing inflammatory pain [3,4].

Tissue acidosis, as a potent marker of inflammation, tumor growth, or ischemia, plays an important role in the genesis of unwanted pain and hyperalgesia. The leading role in acidosis recognition belongs to acid-sensing ion channels (ASICs), members of the degenerin/epithelial sodium channel superfamily [4]. Apart from protons, there are a number of endogenous ASIC modulators: isoquinoline alkaloids, serotonin, FMRF-amide-related neuropeptides, dynorphins, agmatine, arcaine, lysophosphatidylcholine, arachidonic acid, spermine and lactic acid [5,6,7].

In mammals, ASIC isoforms (1a, 1b, 2a, 2b, 3, 4) are encoded by genes ACCN1-ACCN4. ASIC subunits are capable of forming functionally active trimeric homomeric or heteromeric complexes in the organism [8]. Intensively expressed in the central and peripheral nervous systems (CNS and PNS), ASICs are involved in both the nociceptive signal generation, which is associated with the local change in pH (ASIC1-, ASIC2-, and ASIC3-containing channels) under normal and at pathological conditions, as well as synaptic transmission during normal neuronal activity in mechanoreception and chemoreception (ASIC1a, ASIC1b, ASIC2a, and ASIC3) [9,10]. Due to the involvement of ASIC1a and ASIC3 channels in physiological and pathophysiological processes, they are potential targets for the development of new analgesics and neuroprotective drugs [10,11,12].

Some non-selective, low-molecular ligands of ASIC channels, acting at micromolar concentrations, are enabled for clinical medicine and veterinary procedure: local anesthetics (lidocaine and tetracaine), nonsteroidal anti-inflammatory drugs (NSAIDs) (ibuprofen, aspirin, and diclofenac), potassium-sparing diuretic amiloride and the antibacterial/antiparasitic drug, diminazene. The most investigated ligand, amiloride, inhibits ASIC1a, ASIC1b, ASIC2a and ASIC3 channels (IC_50_ 5–100 μM), and potentiates ASIC3/1b and sustains the current of ASIC3 (EC_50_ 560 μM) [7,13]. Preclinical and clinical studies of amiloride have shown the effectiveness of ASIC channel inhibitors as neuroprotective and analgesic drugs [13,14].

Venom-derived toxins affecting ASICs may cause pain or, on the contrary, have analgesic, anti-inflammatory or neuroprotective effects. The first identified peptide agonist of ASIC channels, from the venom of the coral snake *Micrurus tener tener*, is a complex of MitTxα (Kunitz fold) and MitTxβ (phospholipase A2-like protein) subunits [15]. MitTxα/β induces a nociceptive response in mice (paw licking), primarily due to the activation of ASIC1a channels. It also activates ASIC1a, ASIC1b (EC_50_ 9 and 23 nM, respectively) and ASIC3 (EC_50_ 830 nM) channels, and potentiates proton-induced currents through ASIC2a (EC_50_ 75 nM) [15]. Neuropeptide CNF-Tx1.1, from the cone snail *Conus textile*, increases the acid-induced transient muscle pain and chronic hyperalgesia mediated via ASIC3 [16].

Peptide Hi1a (double-inhibitor cystine knot), from the spider *Hadronyche infensa*, demonstrates a pronounced long-time window for neuroprotection after stroke, and is the most active known inhibitor of ASIC1a channels (IC_50_ 0.4 nM) [17]. Protection by Hi1a was effective, even eight hours after a stroke [17], when homologous toxin PcTx1 was ineffective [18,19]. PcTx1 (ICK-peptide), from the tarantula *Psalmopoeus cambridgei*, inhibits ASIC1a (IC_50_ 0.3–3.7 nM [20]) and ASIC1a/2b [21,22], and has a strong antinociceptive effect in the acute and neuropathic pain models [23].

Mambalgins (three-finger toxins), from the venom of the black mamba *Dendroaspis polylepis polylepis* (Ma-1 and Ma-2) and the green mamba *Dendroaspis angusticeps* (Ma-3), have a significant analgesic effect on acute heat and neuropathic pain, and prevent inflammation-induced thermal and mechanical hyperalgesia through the inhibition of ASIC1a- (IC_50_ 3–55 nM [20]) and ASIC1b-containing channels [24,25].

Peptide toxins of sea anemones have been extensively studied for over 40 years [26]. Currently, they can be generally classified into enzymes, cytotoxins and neurotoxins, which include dozens of structural classes [27,28,29]. The most studied among them are actinoporins [30], neurotoxins [31] and Kunitz-type peptides [28,29]. Nevertheless, there are a number of much less studied groups, particularly twisted β-hairpin toxins and APETx-like peptides, which have different activities, including ASIC modulation.

A sea anemone toxin, Ugr 9-1 (twisted β–hairpin), from *Urticina grebelnyi*, as well as a synthetic analog, Ugr22, created on the Ugr 9–1 sequence base, induce an analgesic effect in the inflammation-induced thermal hyperalgesia and acid-induced muscle pain models through the inhibition of both the peak and sustained current of ASIC3 (IC_50_ 10 μM) [32,33].

A toxin, APETx2 (defensin-like), from *Anthopleura elegantissima*, induces antihyperalgesia in an acid-induced muscle pain model [34], prevents acid-induced mechanical hyperalgesia [35], as well as thermal and mechanical hyperalgesia induced by inflammation (both acute and chronic) [34,35,36], partially reduces edema [34] and has an analgesic effect in models of postoperative pain [37] and migraine [38] in rodents. The biological effect of APETx2 is due to the inhibition of nociceptive channels ASIC3 (IC_50_ 37–63 nM [20]) and Na_V_1.8, but the potentiation of ASIC1b and ASIC2a channels, accompanied by its ability to inhibit hERG channels, potentially complicates the use of high doses of APETx2 for clinical practice [36].

Thus, all tested inhibitors of ASIC1a and ASIC3 channels demonstrate an analgesic effect in the inflammation-induced thermal hyperalgesia and acid-induced muscle pain models. In this research, we continued the study of two APETx-like peptides from sea anemone *Heteractis magnifica* (*=Heteractis crispa* [39]), Hmg 1b-2 (=Hcr 1b-2) and Hmg 1b-4 (=Hcr 1b-4), which have been recently shown to inhibit rASIC1a at micromolar concentrations (IC_50_ 4.8 and 1.25 μM, respectively) [40,41]. In addition, Hmg 1b-2 inhibits rASIC3 (IC_50_ 15.9 μM) currents, as well as affects voltage-gated channels and acetylcholine receptors [39,40,41,42], also demonstrating analgesic activity in an acid-induced muscle pain model [40], while Hmg 1b-4 is an enhancer of a proton-evoked rASIC3 current (EC_50_ 1.53 μM) [41], which is likely to make its in vivo effects ambiguous.

Here, we first study the native Hmg 1b-2 and recombinant Hmg 1b-4 activity on human ASIC3-Δ20 channels (hASIC3 truncated by 20 C-terminal amino acid residues), expressed in *Xenopus laevis* oocytes. Moreover, we demonstrate the anti-inflammatory, analgesic and anxiolytic activity of these peptides in vivo.

## 2. Results

### 2.1. Obtaining Recombinant Hmg 1b-4

**Figure 1:**
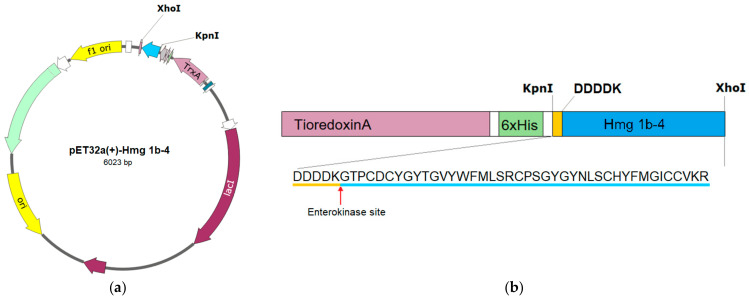
(**a**) Map of the pET32a(+)-Hmg 1b-4 expression plasmid. The sequence encoding the Hmg 1b-4 and enterokinase site was introduced using the restriction enzyme recognition sites KpnI and XhoI. (**b**) The schematic representation of the expected fusion proteins Trx-Hmg 1b-4 and Hmg 1b-4 sequences are shown.

Limited by quantity, peptide Hmg 1b-4 cannot be obtained from a natural source, so its production was organized via protein expression in prokaryotic cells. Plasmid pET32a(+) was chosen as a vector for the expression of the recombinant Hmg 1b-4 peptide, because the fused thioredoxin domain facilitates proper closure of the cysteine residues in the target peptide. The details of the vector construction are shown in Figure 1. The final fusion protein, Trx-Hmg 1b-4, consisted of the target peptide sequence at the C-terminal part following the DDDDK sequence, which allows for specific hydrolysis by enteropeptidase. The 6His affinity tag and the thioredoxin fragment were located at the N-terminal. The expression of the chimeric protein gene was carried out in Shuffle cells for 18 h at room temperature. After cell lysis and metal chelate affinity chromatography, the yield of the fused protein was 15 mg per liter of medium (via spectrophotometric estimation).

**Figure 2:**
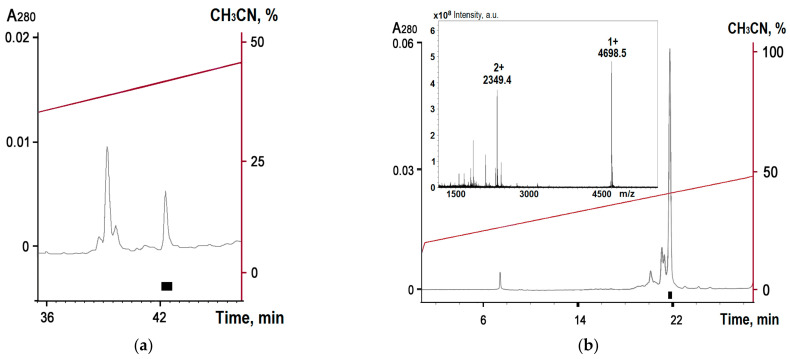
Purification of the toxin after fusion protein digestion by enteropeptidase by RP-HPLC (**a**) on a Jupiter C5 column (150 mm × 4.6 mm) in the presence of 0.1% TFA, and a linear gradient from 0 to 60% MeCN over 60 min at a flow rate 1 mL/min; and (**b**) on a Luna Phenyl–Hexyl column (250 mm × 4.6 mm) in the presence of 0.1% TFA, and at a linear gradient from 20 to 50% over 30 min at a flow rate of 1 mL/min. Black box indicates the elution time of the toxin. The inset shows MALDI analysis of the pure toxin.

The folding of Trx-Hmg 1b-4 in prokaryotic cells was difficult, as with other APETx2-analogous toxins [43]. To obtain a proper protein structure, the refolding with a glutathione pair was carried out for five days at a temperature of 14 °C. The target peptide was isolated after specific hydrolysis by enteropeptidase using RP-HPLC (Figure 2). Mass spectrometry analysis confirmed the formation of disulfide bridges (the molecular weight being 4697.9 Da vs. theoretical 4697.5 Da). The yield of the recombinant Hmg 1b-4 was 0.3 mg per liter of medium.

### 2.2. Electrophysiological Effects of Peptides on ASIC1a and ASIC3 Channels

**Figure 3:**
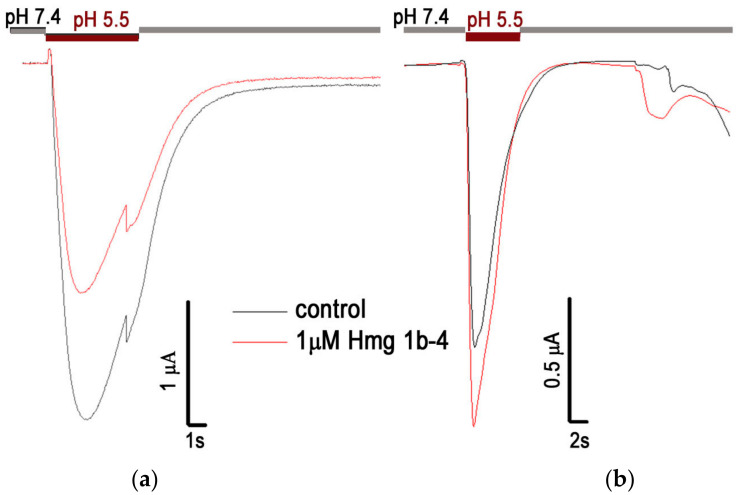
Electrophysiological study of recombinant Hmg 1b-4 on rASIC1a (**a**) and rASIC3 (**b**) channels expressed in *X. laevis* oocytes. Current traces generated by a pH drop of 7.4 to 5.5 are shown for the control application (black line) and the cells pre-incubated with 1 µM of Hmg 1b-4 (red line).

The purified recombinant Hmg1b-4 had the same ability as the native peptide to modulate rat ASICs, expressed in *Xenopus laevis* oocytes, in two-electrode voltage clamp electrophysiological studies. Thus, the application of 1 μM Hmg 1b-4 led to the inhibition of the rat ASIC1a (rASIC1a) currents by 45% (n = 5) and potentiation of the rat ASIC3 (rASIC3) currents by 29% (n = 5) when the current was generated by a drop in the pH from 7.4 to 5.5 (Figure 3). These data were in good agreement with the activity of the native toxin in relation to the same ASIC isoforms [41].

**Figure 4:**
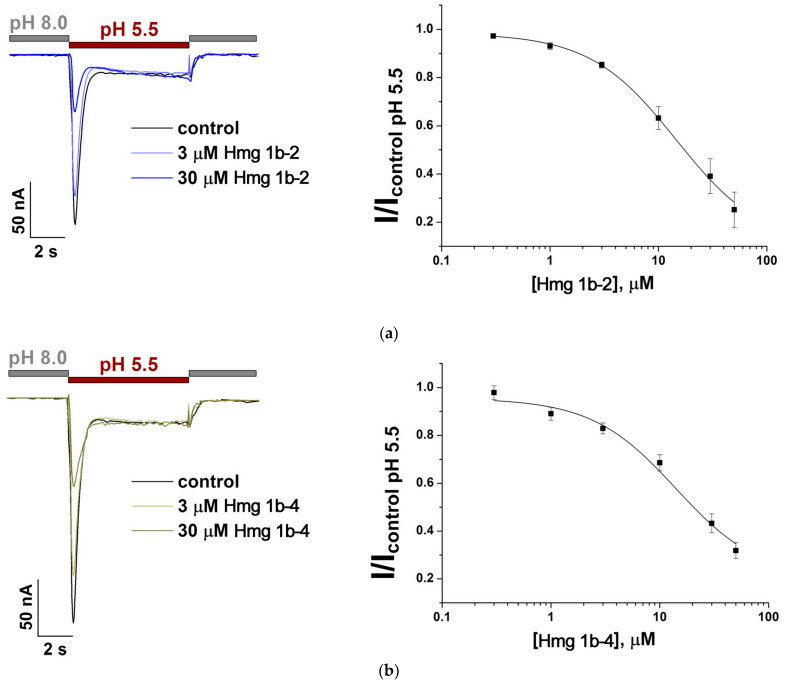
Inhibitory activity of peptides Hmg 1b-2 (**a**) and Hmg 1b-4 (**b**) toward hASIC3-Δ20. Acid-induced currents were evoked by a pH drop from 8.0 to 5.5. Representative current traces (**left panel**) and the concentration–response curve (**right panel**) for the inhibitory effect on the transient current are presented. Data are fitted using the Hill equation, and the resulting values of the fitting parameters are IC_50_ 14.6 ± 0.9 µM (n_H_ of 1.09 ± 0.06) and IC_50_ 14.1 ± 2.5 µM (n_H_ of 1.1 ± 0.2) for Hmg 1b-2 and Hmg 1b-4, respectively. Each point indicates the means ± SEM (n = 5).

To compare the HmgTx mode of action on rat and human ion channels, we measured the effect of recombinant Hmg 1b-4 and native Hmg 1b-2 on human ASIC3 channels truncated by 20 C-terminal amino acid residues (hASIC3-Δ20). This mutant retains the extracellular structure of hASIC3, including all binding sites for activators and modulators, but has a more pronounced transient current after activation [44]. Hmg 1b-2 and Hmg 1b-4 were shown to have approximately the same inhibitory effect on the transient current component of hASIC3-Δ20 expressed in *X. laevis* oocytes. The IC_50_ values were 14.6 ± 0.9 µM and 14.1 ± 2.5 µM, respectively, and did not have any significant effect on the sustained component (Figure 4). Therefore, the mechanism of action of Hmg 1b-4 between human and rat ASIC3 was found to be different.

### 2.3. Animal Experiments

#### 2.3.1. Acute Toxicity

The maximal nontoxic dose of Hmg 1b-2 and Hmg 1b-4 at intraperitoneal administration was identified as 10 mg/kg [40]. Here, we evaluated the toxicity of Hmg 1b-2 and Hmg 1b-4 administrated intravenously to mice at a dose of 1 mg/kg. Peptides did not cause the symptoms of neurotoxicity or obvious changes in the behavior of animals (see open field and elevated plus maze test results below), as with the saline administrated to the control group.

A direct Hmg 1b-4 injection in the hind paw pad did not induce a nociceptive reaction, which may be expected for the rASIC3 potentiator. Thus, a dose of 1 mg/kg was considered the safe option for further experiments.

#### 2.3.2. Behavioral Tests

##### Open Field Test

**Figure 5:**
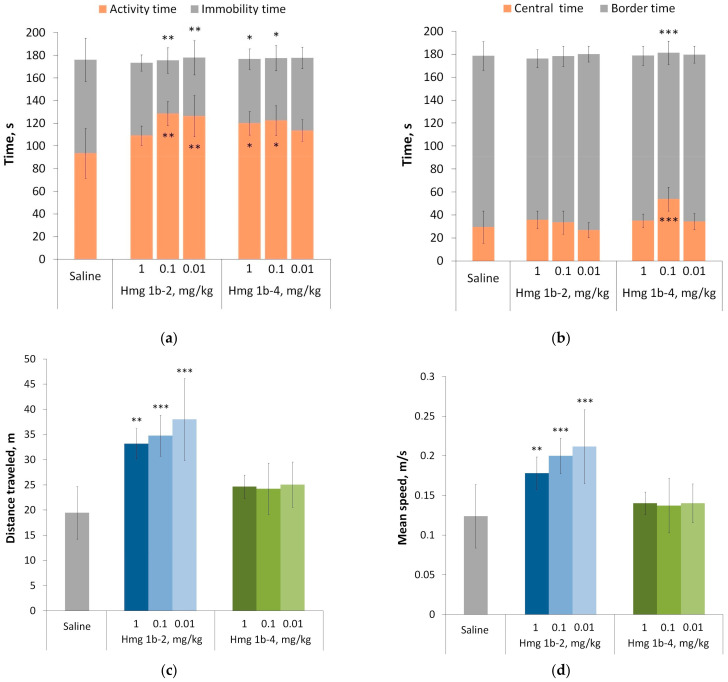

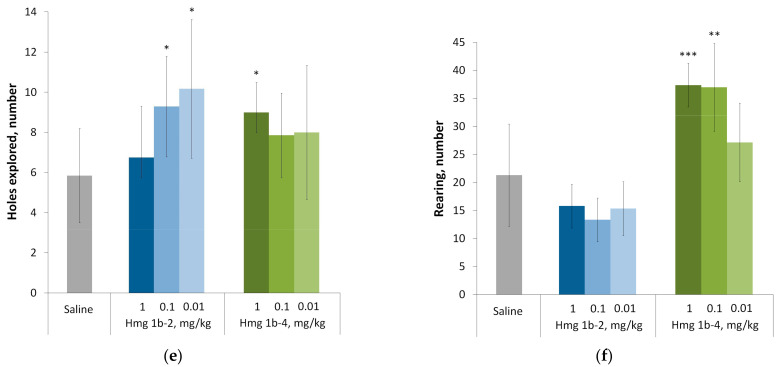
Effect of Hmg 1b-2 and Hmg 1b-4 administered intravenously on normal mouse behavior in the open-field test: time of mouse activity or immobility (**a**), time spent in the central or border zone (**b**), distance traveled by animals (**c**), mean movement speed of animals (**d**), peeps into the holes (**e**) and number of vertical stances (**f**). Control animals received saline in identical volumes. Results are presented as the mean ± SD (n = 7–8). The significance of differences was estimated via a one-way ANOVA, followed by Tukey’s test versus saline group, with * *p* < 0.05, ** *p* < 0.01 and *** *p* < 0.001.

To exclude the possible neurotropic effect of the peptides Hmg 1b-2 and Hmg 1b-4 and evaluate their effect on the mice motor, an orienting–exploratory activity open field test was carried out. The effect of both peptides on the behavior of the mice was unidirectional and expressed as an increase in physical activity, but not as a suppression, due to a possible toxic effect on the CNS. Of the 14 parameters evaluated in the test (Table S1), only those significantly different between any group treated with the peptides and the control group are shown in Figure 5. In the peptide-treated groups, both peptides significantly increased the percentage of the activity time from 50% (in the control group) to 70% (Figure 5a). The peptide Hmg 1b-2 excels Hmg 1b-4 in terms of the travelled distance and speed of movement (Figure 5c,d). Hmg 1b-2 increased the distance travelled and the speed of movement approximately 1.5 times compared with the control group, while Hmg 1b-4 did not affect these options (Figure 5c,d).

In the peptides-treated groups, in addition to greater physical activity, an enhancement in the research behavior was also observed. Both peptides significantly increased the exploration activity of mice, expressed by the increase in the number of peeps into the holes of the experimental arena. The most effective doses for Hmg 1b-2 were 0.1 and 0.01 mg/kg, and 1 mg/kg for Hmg 1b-4 (Figure 5e). Interestingly, in two instances, only Hmg 1b-4, at a dose of 0.1 mg/kg, increased the time the animals spent in the center of the arena compared to the control group (Figure 5b), and the number of vertical stances (Figure 5f) at doses of 0.1 and 1 mg/kg. Both parameters indicated a decrease in the anxiety level of the animals affected by the peptide. The data obtained suggested that Hmg 1b-2 has an excitatory effect on the CNS, but Hmg 1b-4 has an anxiolytic effect.

##### Elevated Plus Maze Test

**Figure 6:**
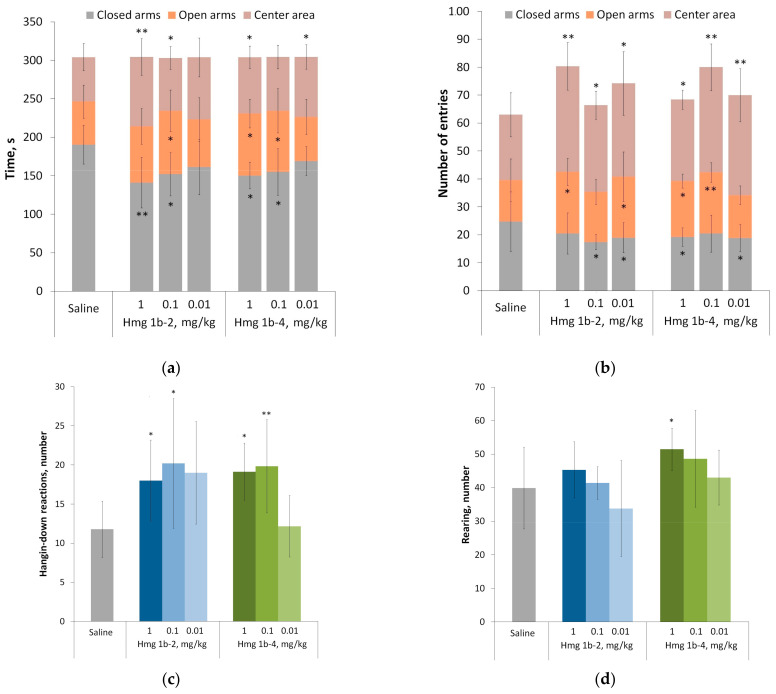
The anti-anxiety activity of Hmg 1b-2 and Hmg 1b-4 administered intravenously in the elevated plus maze test: time spent in the closed arms, open arms and central area (**a**); number of entries to the closed arms, open arms and central area (**b**); number of hanging-down reactions (**c**); and vertical stances (**d**). Control animals received saline in identical volumes. Results are presented as the mean ± SD (n = 7–8). The significance of differences was estimated via a one-way ANOVA followed by Tukey’s test versus the saline group, with * *p* < 0.05 and ** *p* < 0.01.

The anxiolytic potential of the peptides was studied using the elevated plus maze (EPM) test. The test assesses the degree of anxiety resulting from stress caused by height and an uncomfortable environment. The rodent’s fear of heights and reflex preference for a dark enclosed space result in a comfortless exposure within the open and lighted area. As a result of the test, it was shown that the animals of the control group preferred to spend most time in the closed arms of the maze (63% over the total time), which is typical for rodents (Figure 6a). However, the activity of the animals (number of entries into different zones) (Figure 6b) was generally the same as in the groups treated with peptides (30 min before testing). The decrease in the close area preference was reduced to 50% in the peptide-treated groups (Figure 6a). Activity and immobility time, travelled distance and the mean speed were approximately the same in all groups (Table S2). Thus, we conclude that Hmg 1b-2 and Hmg 1b-4 similarly change general motor activity in the EPM test and exhibit an anxiolytic effect.

Some difference in the effect between peptides was found by using a risk assessment parameters calculation. The anxiolytic effect of Hmg 1b-4 over Hmg 1b-2 manifested in an increase in the number of hangings from open sleeves (Figure 6c) and the rears number (Figure 6d). In animals treated with Hmg 1b-2 at doses of between 0.1–1 mg/kg, anxiety was reduced significantly in comparison to the control group, as demonstrated by the number of hangings. At the same time, there was no noticeable difference in the number of times the animals peeked out of closed sleeves between all groups. The results obtained indicate that a balance between exploratory motivation and fear shifted toward the desire to explore after the injection of both peptides.

#### 2.3.3. Analgesic Activity

To assess the analgesic activity of the peptides, a test based on the counting of the number of writhings, a somatosensory reaction for the mice, was used. The writhings were caused by the chemical irritation of the serous membranes after the intraperitoneal administration of acetic acid. This model is widely used to study the ASIC channel modulators involved in pain signal transmission via tissue acidosis [9,13,18]. A nonsteroidal anti-inflammatory drug, diclofenac, was used as a positive control. Diclofenac is a major commercial drug used against inflammation and pain. It inhibits the sustained current component of ASIC3 with the IC_50_ of 92 ± 19 μM, but not the transient component, and decreases the inflammation-induced expression of ASICs [45].

**Figure 7:**
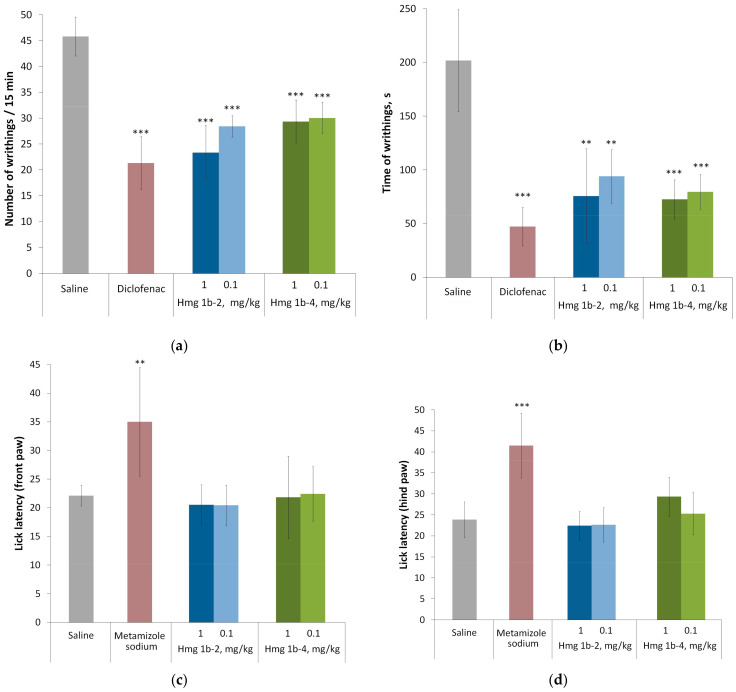
Analgesic activity of Hmg 1b-2 and Hmg 1b-4 administered intravenously in the model of the acid-induced muscle pain: number of writhings (**a**), time of writhings (**b**); and the hot plate test: latent time of front paw licking (**c**), latent time of hind paw licking (**d**). A saline buffer as a negative control and diclofenac at a dose of 1 mg/kg as a positive control were administered intravenously, and metamizole sodium at a dose of 500 mg/kg as a positive control was administered intraperitoneally. Results are presented as the mean ± SD (n = 7–8). The significance of differences was estimated via a one-way ANOVA followed by Tukey’s test versus the saline group, with ** *p* < 0.01 and *** *p* < 0.001.

Both Hmg 1b-2 and Hmg 1b-4, similar to diclofenac, reduced the number of writhings 2 and 1.5 times, respectively, and the duration of writhings by more than 2.5 times vs. the control, and had no significant difference in analgesic activity between each other (Figure 7a,b).

To assess the effect of the peptides on acute pain sensitivity during thermal exposure, the hot plate test was carried out. Metamizole sodium was used instead of diclofenac, since the latter did not affect the pain sensitivity in this test. Metamizole, also known as dipyrone, is a widely used non-opioid analgesic, as well as antipyretic and spasmolytic agent. Its action mechanism does not directly involve ASICs or other ion channels/receptors [46]. Metamizole sodium had a pronounced analgesic effect, increasing the latent time of licking both the front and hind paws more than 1.5 times. The tested peptides did not show a reliable analgesic effect in any of the doses, nor affect the sensitivity of the animals to high temperatures (Figure 7c,d).

#### 2.3.4. Anti-Inflammatory Activity

##### Carrageenan-Induced Paw Edema

The anti-inflammatory activity of Hmg 1b-2 and Hmg 1b-4 was assessed using a model of acute local inflammation induced by λ-carrageenan. In a carrageenan-induced paw edema test, the injection of carrageenan in the hind paw pad of mice showed a time-dependent increase in paw edema. This model is widely used to evaluate the anti-inflammatory effect of potential immunomodulatory and analgesic drugs.

**Figure 8:**
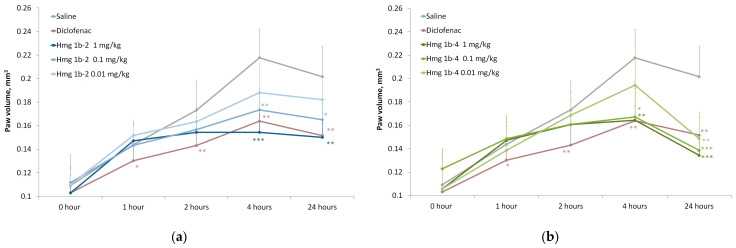

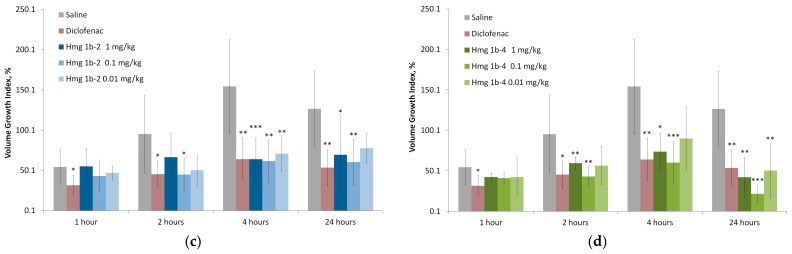
λ-Carrageenan-induced inflammation model. Time-dependent effect of Hmg 1b-2 (**a**,**c**) and Hmg 1b-4 (**b**,**d**) administered intravenously 30 min before the induction of the inflammation on the paw volume (**a**,**b**) and on the volume growth index (%) (**c**,**d**), respectively. A saline buffer as a negative control and diclofenac at a dose of 1 mg/kg as a positive control were administrated intravenously. Results are presented as the mean ± SD (n = 7–8). The significance of differences was estimated via a one-way ANOVA followed by a Tukey’s test versus the saline group, with * *p* < 0.05, ** *p* < 0.01 and *** *p* < 0.001.

The maximum increase in edema was observed in the control group at the fourth hour after carrageenan administration. The peptide Hmg 1b-2 had no significant effect on the paw volume and growth index of inflammatory edema after 1 h and did not show anti-inflammatory activity except for 4 h at a dose of 0.01 mg/kg. However, at doses of 1 and 0.1 mg/kg, Hmg 1b-2 reduced the size of the paw edema by about 1.5 times (after 4 and 24 h) compared to the control group (Figure 8a,c). Diclofenac reduced inflammation more rapidly (1–24 h range), but the effect of Hmg 1b-4 at doses of 1 and 0.1 mg/kg at 24 h exceeded the effect of diclofenac.

The maximal effectiveness in paw edema suppression was exhibited by Hmg 1b-4. It demonstrated a statistically significant anti-inflammatory effect at doses of 0.1 and 1 mg/kg after 2, 4 and 24 h (Figure 8b,d). At 1 and 2 h after the carrageenan administration in the groups treated with the peptide, the paw volume and growth index of the inflammatory edema decreased slightly. However, after 4 h, Hmg 1b-4 at doses of 1 and 0.1 mg/kg reduced the paw edema 1.5 times, and after 24 h, by approximately 2 and 4 times at doses of 1 and 0.1 mg/kg, respectively. At a dose of 0.01 mg/kg, Hmg 1b-4 significantly reduced the volume of the paw edema (by 1.5 times, relative to the control group) only after 24 h.

##### Complete Freund’s Adjuvant-Induced Acute Inflammation

In addition, the anti-inflammatory activity of the peptides at the same concentrations was tested in a model of acute inflammation of the mouse hind paw, induced by the application of the complete Freund’s adjuvant (CFA). CFA causes persistent edema due to the infiltration of lymphocytes and degradation of tissues, primarily joints, cartilage, bone and periosteum [34,47].

**Figure 9:**
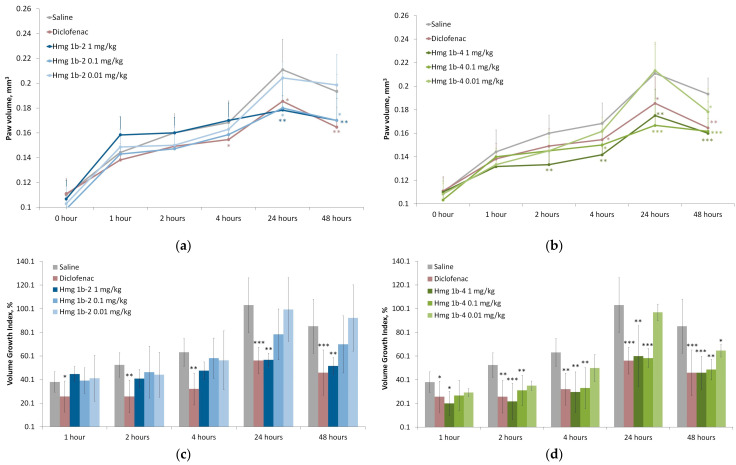
Complete Freund’s adjuvant-induced inflammation model. Time-dependent effect of Hmg 1b-2 (**a**,**c**) and Hmg 1b-4 (**b**,**d**) administered intravenously 30 min before the inflammation induction on the paw volume (**a**,**c**) and the volume growth index (%) (**b**,**d**), respectively. A saline buffer as a negative control and diclofenac at a dose of 1 mg/kg as a positive control were administered intravenously. Results are presented as the mean ± SD (n = 7–8). The significance of differences was estimated via a one-way ANOVA, followed by Tukey’s test versus the saline group, with * *p* < 0.05, ** *p* < 0.01 and *** *p* < 0.001.

In contrast to the carrageenan-induced inflammation model, in the CFA test, Hmg 1b-2 did not provide a significant effect on the rate of inflammatory edema formation (Figure 9a,c). Some reduction at a dose of 1 mg/kg was registered, but only after 24 and 48 h.

Hmg 1b-4 demonstrated an anti-inflammatory effect at all tested doses, and at a dose of 1 mg/kg, the effect of the peptide was more pronounced: the decrease in the paw volume and growth index of the inflammatory edema was more than 1.5 times (Figure 9b,d). The paw edema volume decreasing as a result of Hmg 1b-4 at 1 and 0.1 mg/kg doses was greater than the decreases caused by diclofenac.

## 3. Discussion

Heterologous production of the recombinant hydrophobic APETx-like peptides is challenging, as they possess multiple disulfide bonds of which the proper closure is sensitive to expression conditions and refolding protocol. The technique, successfully applied for obtaining the recombinant APETx2 [43], was useful, but required some changes for the correct folding of Hmg1b-4. Two Met residues in the sequence prevented fusion digestion by cyanogen bromide; therefore, the construction contained thioredoxin domain, six His residues, the DDDDK sequence and the target protein (Figure 1). For this peptide, we used the maximal time of the refolding at a low temperature that allowed shifting the production to the correct recombinant, Hmg 1b-4, with the exact molecular weight and biological activity. Its effects on the rASIC1a and rASIC3 channels (inhibition and potentiation, respectively) were similar to those in previously published data [40,41].

The human ASIC3 channels, activated under the normal conditioning of a pH of 7.4 are known to demonstrate only a pronounced sustained current component. However, this complete loss of the transient current component is restored at a conditioning pH above 7.8 [44]. The intracellular C-terminal domain of hASIC3 channel has been found to be responsible for the decrease in the transient current component [44,48]. For these reasons, in order to demonstrate the effect of HmgTxs on both current components, the hASIC3 truncated by 20 C-terminal amino acid residues (hASIC3-Δ20) with the well-defined transient and sustained component, was used [44]. In two-electrode voltage clamp electrophysiological tests with oocytes expressing hASIC3-Δ20, Hmg 1b-2 and Hmg 1b-4 were shown to have approximately the same inhibitory effect on the transient current component (IC_50_ value for Hmg 1b-2 and Hmg 1b-4 was 14.6 ± 0.9 µM and 14.1 ± 2.5 µM, respectively). These data for Hmg 1b-2 were almost identical to the previously estimated peptide activity toward the rASIC3 with an IC_50_ 15.9 ± 1.1 μM [40]. But for Hmg 1b-4, the effect opposite to rASIC3-potentiating (EC_50_ 1.53 ± 0.07 μM [41]), i.e., inhibition of the human channel was detected.

**Figure 10:**
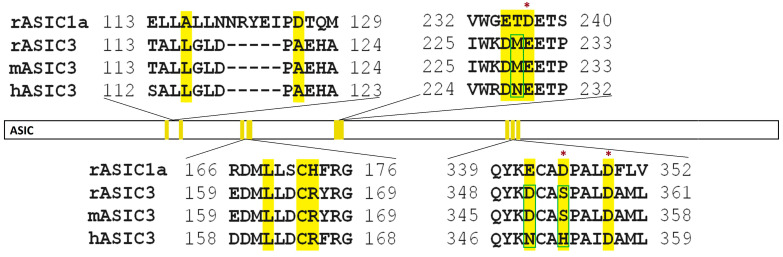
Sequence alignment of rASIC1a (Uniprot ID P55926), rASIC3 (Uniprot ID O35240), mASIC3 (Uniprot ID Q6X1Y6) and hASIC3 (Uniprot ID Q9UHC3). The putative regions involved in the Hmg 1b-4 binding are shown, and the residues that tentatively contribute to the rASIC1a-Hmg 1b-4 complex formation are colored yellow. The residues that distinguished rodent ASIC3 from human ASIC3 are in green boxes, and three proton-sensing residues are marked with red asterisks.

According to the evaluable literature data, the difference in the peptide modulator effect on various ASICs (isoform or species specificity) may be due to two reasons: substitutions within the ligand binding site or substitutions affecting the channel pharmacology, particularly the sensitivity to protons [20,49]. While rat and mouse ASIC3 are extremely close to each other, the human ASIC3 shares only 84% sequence identity with the rat ASIC3. According to previous modeling data [41], Hmg 1b-4 may interact with the acidic pocket of rASIC1a, and the following residues could also be involved: Ala116, Asp126, Leu169, Cys172, His173, Glu235, Thr236, Asp237, Glu342, Asp345 and Asp 349 (Figure 10). Among them Asp237, Asp345 and Asp349 (rASICa numbering) are proton-sensing residues, and Asp237 constitutes a proton-mediated pair with Asp349 to stabilize the open state. There are three substitutions in the proton-binding position that differ from the putative interaction regions of the rodent ASIC3 and the human ASIC3: Met/Asn, Asp/Asn, and Ser/His (Figure 10). Thus, it is clear that the putative binding site of hASIC3 differs to rASIC3. Previously, these channels have not shown identical responses to other ligand administration in electrophysiological experiments and in vivo [20].

To understand the practical potential of the peptides, their activity in behavioral, analgesic and anti-inflammatory mouse models was estimated.

In both open field and EPM tests, Hmg 1b-2 and Hmg 1b-4 administrated intravenously did not demonstrate any toxic or depressing effects on mice. In the case of the animal groups treated with Hmg 1b-4, there was a moderate increase in the activity time, the number of vertical stances and peeps into the holes of the experimental arena compared with the control group. These facts suggest that the increase in animal activity is due to the stimulation of exploratory motivation and active search behavior, but not excitability. An increase in vertical and mink activity in animals under the action of the peptide also indicates an anti-anxiety and possible neuroprotective effect, since stress resistance correlates with quantitative indicators of orienting–exploratory behavior. The influence of Hmg 1b-2 on animals was slightly different; the peptide increased the speed of movement and mink activity. At the same time, vertical activity was reduced, which indicates the possible CNS excitability action of the peptide.

According to the data obtained, Hmg 1b-4 probably provides a more pronounced anxiolytic effect via ASIC1a modulation, since ASIC1a and ASIC2 have been known to express well in CNS. The excitatory effect of Hmg 1b-2 may be partially mediated by its activation effect on Kv1.1 and Kv1.2 channels [42], which also express in the CNS [50]. Contradictory results concerning the involvement of ASIC1a channels in the antidepressant activity and the in vivo effects of ASIC1a inhibitors, namely tarantula toxin, PcTx1, have been published earlier. Thus, in a number of experiments, it was shown that ASIC1a activation leads to the development of anxiety, and the use of channel inhibitors reduces such manifestation [51,52]. However, other data show that ASIC1a activation reduces anxiety-like behavior, while inhibition by PcTx1 increases anxiety levels in rats [53]. These discrepancies are explained by the disparity in the animal species used for in vivo experiments, ASIC1a activity in various brain regions, or by differences in the contribution of ASIC1a to innate and acquired fear [54]. Interestingly, ASIC3 inhibitors, APETx2 and Ugr 9-1, administrated intramuscularly at a dose of 1 mg/kg did not impair locomotion and the CNS-mediated behavior in the open field test [34].

The acid sensitivity of nociceptors is associated with the activation of the H^+^-dependent ion channels, such as ASICs, but also the polymodal transient receptor potential cation channel subfamily V member 1 (TRPV1) [20]. In an acid-induced muscle pain model directly related to the activation of ASICs, Hmg 1b-2 and Hmg 1b-4 administered intravenously, especially at a dose of 1 mg/kg, demonstrated potent analgesic activity by reducing the number and duration of writhings. In the hot plate test, which most objectively reflects TRPV1 channel activation by heat, none of the peptides were active. Thus, it can be concluded that the analgesic effect of the peptides is due to their combined action on the two major ASIC isoforms of PNS, the ASIC1a and ASIC3 channels [10], but not TRPV1.

During pathophysiological conditions such as inflammation, in rodent models, local extracellular pH can decrease physiological values (generally about 7.4) to 6.8 [55,56,57,58], causing an increase in ASIC transcript levels in vivo. In the model of acute local inflammation induced by λ-carrageenan, Hmg 1b-4 had a more pronounced and statistically significant anti-inflammatory effect than its homologue Hmg 1b-2. At doses of 1 and 0.1 mg/kg at 24 h, Hmg 1b-4 exceeded the effect of diclofenac and at dose of 0.1 mg/kg, reduced the volume of the paw almost to the original values.

In the acute local CFA-induced inflammation model, Hmg 1b-4 demonstrated an anti-inflammatory effect at all tested doses, while at a dose of 1 mg/kg, its effect was more pronounced, and at a dose of 1 mg/kg, it decreased the paw edema volume better than diclofenac. The peptide Hmg 1b-2 did not have a significant effect on the rate of inflammatory edema formation. These data lead us to believe that Hmg 1b-4 is now the most effective anti-inflammatory APETx-like peptide from sea anemones, since it is significantly superior not only to Hmg 1b-2, but also to APETx2 and Ugr 9-1 [34].

Since we observed a different effect of Hmg 1b-4 on expressed rASIC3 and hASIC3, the in vivo effects on humans may be also different. This forewarning must be taken into account if the peptides will be promoted for drug seeds. In addition, further verification is required for the effects of Hmg 1b-2 and Hmg 1b-4 activity in the expression system on mammalian cells, since there are cases of the lack of peptide activity on mammalian cells compared to *Xenopus* oocytes [20].

## 4. Materials and Methods

### 4.1. Obtaining Peptides Hmg 1b-2 and Hmg1b-4

The major compound of crude sea anemone extract, Hmg 1b-2, was isolated from the 70% aqueous ethanolic extract of *Heteractis magnifica* in accordance with methods [40], without modifications. The minor compound, Hmg 1b-4, was obtained as a recombinant analog after its expression in competent *Escherichia coli* Shuffle cells. The cells, transformed by pET32a(+) with target genes (Hmg 1b-4) (JSC Eurogen, Moscow, Russia) fused with the thioredoxin domain, were grown in an LB medium containing ampicillin (100 μg/mL), at moderate aeration, stirring and a temperature of 37 °C, to an optical density of OD_600_ 0.6–0.8. The expression of the fusion protein was induced by adding isopropyl 1-thio-β-D-galactopyranoside (IPTG) to a final concentration of 0.2 mM. After 16 h, the cells were harvested via centrifugation for 10 min at 6000 rpm. The cells were resuspended in a buffer for metal affinity chromatography containing 400 mM NaCl and 20 mM Tris-HCl (pH 7.5), with the addition of glycerin up to 50% concentration. Cell lysis was performed using ultrasonic disintegration. The resulting lysate was purified via centrifugation at a speed of 9000 rpm for 10 min at 4 °C.

The affinity chromatography on a HisPur™ Ni-NTA Resin column (Thermo scientific, Waltham, MA, USA) was performed using a 500 mM NaCl, 20 mM Tris-HCl (pH 7.5) and 250 mM imidazole elution buffer. After dilution to a concentration of 1 mg/mL a recombinant product was refolded in the presence of 4 mM reduced (GSH) and 1 mM oxidized (GSSH) forms of glutathione. The refolding was carried out at a temperature of 14 °C and stirring for 72–84 h, followed by desalting via RP-HPLC on a Jupiter C5 column (Phenomenex, Torrance, CA, USA) and lyophilization. Then, the fusion was dissolved to a concentration of 1 mg/mL with a buffer solution containing 5 mM CaCl_2_ and 50 mM TRIS-HCl (pH 8.0), and digested by enteropeptidase (0.8 μg to 1 mg of fusion protein) at 30 °C and stirred for 16–24 h.

The purification of Hmg1 b-4 was carried out using RP-HPLC in two stages: on a Jupiter C5 column (150 mm *×* 4.6 mm, Phenomenex, USA) using flow rate of 1 mL/min and a linear gradient of acetonitrile concentration from 0 to 60% in the presence of 0.1% TFA over 60 min, and on a Luna Phenyl–Hexyl column (250 mm × 4.6 mm, 5 μm, Phenomenex, USA) using a flow rate of 1 mL/min and a linear gradient of acetonitrile concentration from 0 to 60% in the presence of 0.1% TFA over 60 min.

The peptide molecular weight was identical to the calculated one, which was confirmed via matrix-assisted laser desorption ionization (MALDI) using an Ultraflex TOF/TOF mass spectrometer (Bruker Daltonik, Karlsruhe, Germany). 2,5-Dihydroxybenzoic acid (Sigma-Aldrich, Saint Louis, MO, USA) was used as a matrix.

### 4.2. Electrophysiology

Electrophysiological measurement was carried out using the method of two-electrode fixation of the membrane potential on the oocytes of *X. laevis*. Oocytes without a follicular membrane were injected with 2.5 ng of ASIC1a mRNA or 10 ng of ASIC3 mRNA from rat or human, and incubated for 2–3 days at a temperature of 17–19 °C in an ND-96 medium (96 mM NaCl, 5 mM HEPES, 2 mM KCl, 1.8 mM CaCl_2_, 1 mM MgCl_2_ (pH 7.4)) containing gentamicin (50 μg/mL). The membrane potential was fixed at −50 mV; the filter data were recorded at a frequency of 10 Hz and digitized at a frequency of 100 Hz. The data were recorded and digitized using the GeneClamp 500 amplifier (Axon Instruments, Burlingame, CA, USA). An in-house program controlled the synchronized delivery of solutions through the chamber and the recording of the results of the electrophysiological tests. An ND-96 solution (pH 7.4) was used as a conditioning buffer. In the case of human ASIC3, a conditioning buffer with a pH of 8.0 was used. The dry sample under study was dissolved in ND-96 with the appropriate pH value to the desired concentration before the test. The activation of the channels was carried out by a change in the pH of the solution to 5.5 due to the rapid replacement of the solution in the chamber, in which 10 mM MES (pH 5.5) was used as a buffer instead of 5 mM HEPES (or 5 mM HEPPS in the case of conditioning pH 8.0). The application of the peptides was carried out 15 s before changing the conditioning to activate the buffer. The concentration-dependent decrease in the current amplitude through hASIC3, in response to a pH 5.5 stimulus after peptide application, was fitted using the Hill equation: y = ((A1 − A2)/(1 + ([C]/IC_50_)^nH^)) + A2, where y is the relative value of current inhibition, A1 is the control response value, C is the peptide concentration, IC_50_ is the half-maximal inhibitory concentration, n_H_ is the Hill coefficient and A2 is an amplitude of maximum inhibition (% of control).

### 4.3. Animal Studies

The animal studies were performed under the European Commission’s legislation (Directives 86/609/EEC, 2010/63/EU), the National Standard of the Russian Federation “Good Laboratory Practice” (GOST P 53434-2009, Moscow, Russia), and Committee on Ethics of Laboratory Animal Handling № 05/21, 20 September 2021 and 08/21, 30 December 2021 protocols (PIBOC FEB RAS). Adult female CD-1 mice weighing 25 ± 2 g were subjected to a 12 h light–dark cycle at room temperature and with ad libitum access to food and water. There were seven or eight individuals in each group.

#### 4.3.1. Acute Toxicity

Lyophilized peptides were dissolved in 0.9% NaCl solution (saline) and administered once intravenously at doses of 1 mg/kg; the control group received saline (10 mL/kg or 250 µL/mouse). Then, alterations in physiological parameters, such as motility, behavioral responses and physical activity, were registered in each of the animal groups over 24 h.

For a direct Hmg 1b-4 injection (to eliminate the nociceptive reaction of potentiator of ASIC3 channels), it was dissolved in saline and administered at a dose of 1 mg/kg in the hind paw pad. The control group was injected with an identical volume (20 µL) of saline. The animals were placed individually, and a nociceptive reaction was registered for 15 min.

#### 4.3.2. Open Field Test

The animal activity and anxiety-related and exploratory behaviors were estimated at the open field facility (OpenScience, Krasnogorsk, Russia). Three groups of animals were treated with peptides at doses of 0.01, 0.1 and 1 mg/kg (intravenously) 30 min before testing. The control group was injected with an identical volume (50 µL) of saline. The animals were placed in the center of the lightened arena, and the recording lasted 3 min. Using the software “Minotaur” (LLC “Neurobiotics”, Zelenograd, Russia), 14 parameters were estimated: time (T) of activity (act); passivity (pass); spent on the central zone (c.z.), staying on the border zone (b.z.); exit from the central zone (e.c.z.); average travel speed (V, m/s); average movement speed during activity (V(act)); distance traveled (S, m); distance traveled during activity (S (act)); number (N) of visits to central zone (c.z.); visits to side platform (s.p.); vertical activity (racks); peeps into minks (peeps); and bowel movements (def).

#### 4.3.3. Elevated Plus Maze Test

The anti-anxiety activity of the peptides was studied using the elevated plus maze (OpenScience, Krasnogorsk, Russia) test, a cruciform arena raised 50 cm above the floor, where the central platform and two arms are open and lightened, and two arms are closed with walls. Three groups of animals were treated with peptides at doses of 0.01, 0.1 and 1 mg/kg (intravenously) 30 min before testing. The control group was injected with an identical volume (50 µL) of sterile saline. Animals were placed in the center of the lightened arena and the recording lasted 5 min. Using the software “Minotaur” (LLC “Neurobiotics”, Zelenograd, Russia), 12 parameters were estimated: time (T) of activity(act); passivity (pass); spent in closed, open arms, and on the central area; exit from the central zone (e.c.z.); average travel speed (V, m/s); average movement speed during activity (V(act)); distance traveled (S, m); distance traveled during activity (S (act)); a number (N) of entries into open, closed arms, and exits to the central area (entries); vertical activity (racks); and stretch–attend posture when the animal stretches to its full length mainly using the forepaws (sap).

#### 4.3.4. Acetic-Acid-Induced Writhings

Three groups of animals were treated with peptides at doses of 0.01, 0.1 and 1 mg/kg (intravenously) 15 min before an injection of 300 μL of a 0.7% acetic acid solution. The control animal group received an identical volume of saline. The positive control group received intravenously a dose 1 mg/kg of diclofenac (Hemofarm A.D., Vršac, Serbia), a commercial drug of the NSAID group. Fifteen minutes immediately after acid administration, the number of writhings and the writhing time (time in a spasmodic state) were counted.

#### 4.3.5. Hot Plate Test

Thermal analgesia was tested with a Hot Plate Analgesia Meter (IITC Life Science Inc., Woodland Hills, CA, USA). Measurements were carried out 15 min after the intravenous injection of peptides at doses of 0.1 and 1 mg/kg. The control animals received 300 µL of sterile saline. Metamizole (Pharmstandard, Russia) was used as a positive control administrated intraperitoneally at a dose of 500 mg/kg [59] 30 min before testing. Animals were placed individually on the preheated surface of a plate (up to 52 °C), and nociceptive reactions, withdrawal, or licking of the front and hind legs were recorded. The maximum time the animal stayed on the plate did not exceed 60 s.

#### 4.3.6. Carrageenan-Induced Paw Edema

Three groups of animals were treated with peptides at doses of 0.01, 0.1 and 1 mg/kg (intravenously) 30 min before the induction of inflammation. The control animal group received an identical volume of saline. The positive control group received diclofenac (Hemofarm A.D., Vršac, Serbia) at a dose of 1 mg/kg. The edema was induced by a 20 µL injection of 1.5% solution of λ-carrageenan (Sigma Aldrich, St. Louis, MO, USA) in the hind paw pad. The resulting edema was measured after 1, 2, 4 and 24 h using a plethysmometer (Ugo Basile, Gemonio (VA), Italy). The anti-inflammatory threshold was estimated as a decrease in the paw volume and volume growth index (%), calculated using the formula: Volume Growth Index (%) = [(V_C_ − V_i_)/V_i_] × 100, (1), where V_C_ is the volume of the paw after the introduction of carrageenan and V_i_ is the volume of the paw before the introduction of carrageenan.

#### 4.3.7. Complete Freund’s-Adjuvant-Induced Acute Inflammation

Three groups of animals were treated with peptides at doses of 0.01, 0.1 and 1 mg/kg (intravenously) 30 min before the induction of inflammation. The control animal group received an identical volume of saline. The positive control group received diclofenac (Hemofarm A.D., Vršac, Serbia) at a dose of 1 mg/kg. The edema was induced by a 20 µL injection of complete Freund’s adjuvant (CFA) (MP Biomedicals, Irvine, CA, USA) in the hind paw pad. The resulting edema was measured at 1, 2, 4, 24 and 48 h using a plethysmometer (Ugo Basile, Gemonio (VA), Italy). The anti-inflammatory threshold was estimated as a decrease in the paw volume and volume growth index (%), calculated using the formula: Volume Growth Index (%) = [(V_CFA_ − V_i_)/V_i_] × 100, (1), where V_CFA_ is the volume of the paw after the introduction of CFA and V_i_ is the volume of the paw before the introduction of CFA.

### 4.4. Statistic Calculation

All data are expressed as the mean ± S.D. The one-way analysis of variance (ANOVA), followed by Turkey’s post hoc test, was performed using the OriginPro 8.5 (Origin Lab^®^ Corporation, Northampton, MA, USA) software to determine statistical significance. *p* values less than 0.05 were considered statistically significant.

## Data Availability

Not applicable.

## Supplementary Materials

The following supporting information can be downloaded at: https://www.mdpi.com/article/10.3390/toxins15050341/s1, Table S1: Open field test. Parameters of motor and orienting–exploratory activity of mice treated with Hmg 1b-2 and Hmg 1b-4; Table S2: Elevated plus maze test. Parameters of anti-anxiety activity of mice treated with Hmg 1b-2 and Hmg 1b-4.
