# Supplementary material for: Anxiolytic, Analgesic and Anti-Inflammatory Effects of Peptides Hmg 1b-2 and Hmg 1b-4 from the Sea Anemone Heteractis magnifica
Source: Toxins (Basel). 2023 May 15;15(5):341. doi: 10.3390/toxins15050341 (PMC10221320; doi:10.3390/toxins15050341)
Supplement: Supplementary file 1 [file toxins-15-00341-s001.zip › toxins-2359085-supplementary.pdf]

toxins

MDPI

Table S1. Open field test. Parameters of motor and orienting-exploratory activity of mice treated with Hmg 1b-2 and Hmg 1b-4.

Group	T (act), s	T (pass), s	T (c.z.), s	T (b.z.), s	V, m/s	V(act), m/s	S, m	S (act), m	N (c.z.)	N (s.p.)	N (racks)	N (peeps)	N (def)	T (e.c.z.), s
**Saline**	92.5 ± 22.1	80.5 ± 19.1	27.4 ± 14.0	145.8 ± 12.4	0.12 ± 0.04	0.23 ± 0.05	19.4 ± 5.2	21.8 ± 7.5	11.8 ± 5.5	11.4 ± 5.2	21.3 ± 9.1	5.8 ± 2.3	0.3 ± 0.8	11.4 ± 6.7
**Hmg 1b-2 1 mg/kg**	109.1 ± 8.6	64.2 ± 7.2	34.8 ± 7.7	139.5 ± 7.7	0.19 ± 0.02 **	0.31 ± 0.05	33.2 ± 2.9 **	33.2 ± 3.0	20.3 ± 5.4	21 ± 5.0	15.8 ± 3.9	6.7 ± 2.5	0.2 ± 0.4	5.2 ± 1.9
**Hmg 1b-2 0.1 mg/kg**	128.5 ± 10.7 **	47.0 ± 11.3 **	32.6 ± 10.0	143.80 ± 8.8	0.20 ± 0.02 ***	0.29 ± 0.03	34.7 ± 4.0 ***	36.7 ± 6.8	18.8 ± 5.1	19 ± 4.8	13.3 ± 3.9	9.3 ± 2.5 *	0	4.6 ± 1.3
**Hmg 1b-2 0.01 mg/kg**	126.3 ± 5.5 **	51.6 ± 4.7 **	25.8 ± 6.1	153.3 ± 5.5	0.21 ± 0.01 ***	0.30 ± 0.01	37.9 ± 1.6 ***	37.9 ± 6.4	17 ± 2.1	17.2 ± 2.0	15.3 ± 2.9	10.2 ± 0.7 *	0	4.2 ± 1.8
**Hmg 1b-4 1 mg/kg**	119.9 ± 10.4 *	56.8 ± 9.1 *	34.9 ± 5.9	143.8 ± 8.2	0.14 ± 0.01	0.21 ± 0.02	24.6 ± 2.3	24.6 ± 7.5	16.8 ± 2.7	16.9 ± 2.8	37.4 ± 3.9 ***	9.0 ± 1.5 *	0.5 ± 0.7	6.4 ± 2.4
**Hmg 1b-4 0.1 mg/kg**	122.3 ± 13.1 *	55.1 ± 11.1	53.8 ± 10.3 ***	127.5 ± 10.0 ***	0.14 ± 0.03	0.22 ± 0.03	24.2 ± 5.1	24.2 ± 5.1	21.2 ± 6.1	20.1 ± 6.4	37.0 ± 7.8 **	7.8 ± 2.1	0	6.1 ± 3.3
**Hmg 1b-4 0.01 mg/kg**	113.5 ± 9.5	64.2 ± 7.4	34.2 ± 2.8	145.4 ± 5.2	0.14 ± 0.02	0.22 ± 0.02	25.0 ± 1.8 **	25.0 ± 2.4	12 ± 0.8	13.3 ± 1.1	27.2 ± 1.9	8.0 ± 0.5	1.2 ± 0.2	6.5 ± 3.1

Where T (act)—activity time; T (pass)—passivity time; T (c.z.)—time spent on the central zone, s; T (b.z.)—staying on the border zone, s; V, m/s—average travel speed; V(act), m/s—average movement speed during activity; S, m—distance traveled; S (act), m—distance traveled during activity; N (c.z.)—number of visits to central zone; N (s.p.)—number of visits to side platform; N (racks)—vertical activity, the number of racks; N (peeps)—holes explored, the number of peeps into minks; N (def)—number of bowel movements; T (e.c.z.)—time of exit from the central zone, s. Results are presented as mean ± SD (n = 7–8). The significance of differences was estimated via one-way ANOVA followed by a Tukey's test versus saline group, with * $p < 0.05$, ** $p < 0.01$, and *** $p < 0.001$.

Table S2. Elevated plus maze test. Parameters of anti-anxiety activity of mice treated with Hmg 1b-2 and Hmg 1b-4.

Group	T (act), s	T (pass), s	N. entries			T., s			V, m/s	V(act), m/s	S, m	S (act), m	N (racks)	N (sap)	N (h-d)	T (e.c.z.),
			Closed arms	Open arms	Central area	Closed arms	Open arms	Central area								
**Saline**	**153.3 ± 27.6**	**146.4 ± 27.6**	**25 ± 10.6**	**15 ± 7.7**	**23 ± 7.8**	**193.4 ± 25.3**	**62.9 ± 21.5**	**59.7 ± 17.5**	**0.13 ± 0.02**	**0.23 ± 0.02**	**40 ± 6.9**	**38 ± 7.4**	**40 ± 12.1**	**13 ± 3.1**	**12 ± 3.6**	**10.1 ± 9.3**
**Hmg 1b-2 1 mg/kg**	162.3 ± 21.8	137.6 ± 21.9	20 ± 7.3	22 ± 4.8 *	38 ± 8.5 **	151.1 ± 32.7 **	79.1 ± 23.3	93.5 ± 23.8 **	0.14 ± 0.02	0.25 ± 0.02	42.3 ± 5.8	40.5 ± 5.8	51 ± 6.3 *	13 ± 3.0	19 ± 3.6 *	3.3 ± 2.3
**Hmg 1b-2 0.1 mg/kg**	172.4 ± 19.6 *	127.5 ± 19.8 *	17 ± 2.7 *	18 ± 4.4	31 ± 5.0 *	152.1 ± 28 *	82.1 ± 27 *	68.7 ± 14.8 *	0.14 ± 0.01	0.24 ± 0.01	42.9 ± 4.3	41.1 ± 4.5	49 ± 14.4	15 ± 3.7	20 ± 5.9 *	4.6 ± 1.1
**Hmg 1b-2 0.01 mg/kg**	151.3 ± 7.4	148.5 ± 7.3	19 ± 5.3	23 ± 8.9 *	33 ± 11.3 *	157.2 ± 35.7	60.9±28.3	79.7 ± 25.3	0.13 ± 0.01	0.24 ± 0.02	37.8 ± 2.9	36.3 ± 2.8	43 ± 8.2	17 ± 4.5 *	12 ± 3.9	5.2 ± 4.6
**Hmg 1b-4 1 mg/kg**	174 ± 17.9 *	125.7 ± 17.7 *	19 ± 3.3 *	20 ± 2.5 *	29 ± 3.4 *	160.2 ± 17.2 *	89.6 ± 18.5 *	73.8 ± 14.6 *	0.14 ± 0.03	0.25 ± 0.04	43.1 ± 7.1	41.0 ± 7.5	45 ± 8.4	14 ± 1.8	18 ± 5.2 *	8 ± 6.5
**Hmg 1b-4 0.1 mg/kg**	170.8 ± 16.1	128±15.7	20 ± 6.6	22 ± 3.5 **	38 ± 8.4 **	169 ± 30.2 *	84.3 ± 28.7 *	78.7 ± 15.3	0.14 ± 0.02	0.27 ± 0.03	46.8 ± 6.1	45.1 ± 6.1	41 ± 4.8	17 ± 3.6 *	20 ± 8.3 **	8 ± 3.6
**Hmg 1b-4 0.01 mg/kg**	161.7 ± 10.6	137.7±11.2	18 ± 4.8 *	15 ± 3.3	36±9.4 **	180.1 ± 18.7	69.3 ± 22.8	86.8 ± 15.9 *	0.15 ± 0.03	0.26 ± 0.02	44.3 ± 8.3	42.6 ± 8.0	34 ± 14.3	15 ± 2.3	19 ± 6.6	3 ± 1.5

Where T (act)—activity time; T (pass)—passivity time; N. entries—number of entries; T., s—time spent in closed, open arms and in central area; V, m/s—average travel speed; V(act), m/s—average movement speed during activity; S, m—distance traveled; S (act), m—distance traveled during activity; N (racks)—vertical activity, the number of racks; N (sap)—number of stretch-attend postures, when animal stretches to its full length mainly using the forepaws; N (h-d)—number of hanging-down reactions; T (e.c.z.)—time of exit from the central zone, s. Results are presented as mean ± SD (n = 7–8). The significance of differences was estimated via one-way ANOVA followed by a Tukey's test versus saline group, with * $p < 0.05$, ** $p < 0.01$.
